# Investigation of Parasitic Infection in Crocodile Lizards (*Shinisaurus crocodilurus*) Using High-Throughput Sequencing

**DOI:** 10.3390/ani12202726

**Published:** 2022-10-11

**Authors:** Yongru Zeng, Yi Xiong, Chunsheng Yang, Nan He, Jiasong He, Wenxian Luo, Yaohuan Chen, Xiaochen Zeng, Zhengjun Wu

**Affiliations:** 1 Key Laboratory of Ecology of Rare and Endangered Species and Environmental Protection, Guangxi Normal University, Ministry of Education, Guilin 541004, China; 2 Guangxi Key Laboratory of Rare and Endangered Animal Ecology, College of Life Science, Guangxi Normal University, Guilin 541004, China; 3 Daguishan National Nature Reserve for Crocodile Lizards, Hezhou 542824, China; 4 Guangdong Luokeng *Shinisaurus crocodilurus* National Nature Reserve, Shaoguan 512100, China

**Keywords:** *Shinisaurus crocodilurus*, parasite, high-throughput sequencing, captive breeding

## Abstract

**Simple Summary:**

To explore unexplained crocodile lizard deaths, we analyzed fecal samples from crocodile lizards at the Daguishan and Luokeng nature reserves using high-throughput sequencing to investigate parasitic infections in this endangered species. The present study reported the occurrence of parasitic infection in wild and captive crocodile lizards, with a total parasitic infection rate of 33.33% (23/69). Different influencing factors (populations and regions) were analyzed, and significant differences were found. The results indicate that captive crocodile lizards were more susceptible to parasitic diseases than wild crocodile lizards. In addition, only *Cryptosporidium* infection varied by geographical location, with crocodile lizards from Daguishan showing higher susceptibility to *Cryptosporidium* infestation. From a disease prevention perspective, these findings have great significance for crocodile lizard conservation.

**Abstract:**

The highly endangered crocodile lizard (*Shinisaurus crocodilurus*) continues to be impacted by disease, especially in captive breeding populations. In this paper, based on high-throughput sequencing, we investigated parasitic infections in captive and wild crocodile lizard populations in the Daguishan National Nature Reserve and Guangdong Luokeng *Shinisaurus crocodilurus* National Nature Reserve. The results show that the overall parasitic infection rate in crocodile lizards was 33.33% (23/69). Four parasite genera were detected, including *Eimeria*, *Cryptosporidium*, *Nematopsis*, and *Acanthamoeba*, with infection rates of 15.94% (11/69), 17.39% (12/69), 7.25% (5/69), and 4.35% (3/69), respectively. Significant differences in the infection rate were found between the different parasite species (χ^2^ = 8.54, *p* < 0.05, chi-squared test). The parasitic infection rates in the captive and wild populations were 39.29% (22/56) and 7.69% (1/13), respectively, which were significantly different (*p* < 0.05, Fisher’s exact test). However, no significant differences in the infection rates of the four parasite genera were found between the captive and wild populations (*p* > 0.05, Fisher’s exact test). The parasitic infection rates in Daguishan and Luokeng were 34.09% (15/44) and 32.00% (8/25), respectively, which were not significantly different (*p* > 0.05, Fisher’s exact test). However, significant differences in terms of species were found in the two reserves (*p* < 0.01, Fisher’s exact test). Only *Cryptosporidium* infection showed a significant difference between the two regions (*p* < 0.01, Fisher’s exact test). Our results suggest that captive crocodile lizards are more susceptible to parasitic diseases than wild crocodile lizards and that *Cryptosporidium* infection varies by geographical region. This study provides basic information about the parasites of endangered crocodile lizards, as well as a reference for disease control and conservation.

## 1. Introduction

With the current acceleration of biodiversity loss, it is critical to protect and increase the populations of declining and endangered species [1,2,3]. For endangered animals, establishing captive breeding populations may be an effective conservation strategy [1]. However, the adequate maintenance of captive populations in nature reserves remains challenging [4]. Notably, unknown diseases can have considerable impact on the welfare and health of captive animals and can adversely affect conservation programs aimed at protecting endangered species [5]. Of concern, this issue has occurred in captive crocodile lizards (*Shinisaurus crocodilurus*) within the last five years in China [6].

The relict crocodile lizard (*Shinisaurus crocodilurus* Ahl, 1930) is highly endangered and currently listed on the IUCN Red List of Threatened Species. The species is only found in a few nature reserves in the Guangxi and Guangdong provinces of China and in northern Vietnam. Wild populations have decreased dramatically due to various external factors, such as hunting, habitat destruction, and environmental change, with current estimates of 1200 individual lizards in China [7] and 150 individual lizards in Vietnam [8]. To restore wild populations, captive breeding and release initiatives have been conducted at the Guangxi Daguishan National Nature Reserve and Guangdong Luokeng *Shinisaurus crocodilurus* National Nature Reserve [7]. However, high captive densities, poor husbandry techniques, continual cohabitation, and intense human interactions can increase exposure to pathogens [9,10]. In recent years, captive crocodile lizard populations have been plagued by a variety of unknown diseases, leading to many deaths each year [7]. In particular, two distinct skin diseases have been reported in captive populations. The first is a skin nodular disease caused by *Austwickia chelonae*, which has resulted in the death of 69 crocodile lizards in the Luokeng Reserve [11]. The second is a skin lesion and ulcer disease, likely caused by *Pseudomonas aeruginosa*, which has resulted in the death of more than 30% of juvenile crocodile lizards (aged <1 year) in the Daguishan and Luokeng reserves [6]. Of concern, these skin diseases have also been detected in wild crocodile lizards in Daguishan Reserve [6]. Furthermore, Jiang et al. [12] recently isolated *Morganella morganii* from the liver of a dead juvenile crocodile lizard. These findings suggest that many impactful crocodile lizard diseases may be caused by bacteria, with potential co-infection with other pathogens. However, the species of pathogens that infect crocodile lizards are not fully understood, and the specific causes of death have not yet been adequately investigated.

Bacteria, parasites, and viruses are the most common pathogenic microorganisms. However, it can be easy to overlook parasitic infections as they are frequently chronic in nature. Wild and captive reptiles are vulnerable to parasites both in vitro and in vivo. In Australia, there are more than 70 different species of ticks, 14 of which will parasitize reptiles [13]. Furthermore, *Cryptosporidium* infections have been reported in at least 57 reptilian species [14], with chronic cryptosporidiosis and lethality in some snakes [14,15]. Reptile survival and reproduction can be seriously impacted by parasitic infection, leading to disease and mortality in captive reptiles as well as reduced resistance and increased susceptibility to various diseases [16,17]. Hence, understanding parasitic diseases in crocodile lizards is critical for determining mortality associated with parasitic infection.

Traditional parasite detection requires host dissection, microscopic examination, and morphological identification, which can be time-consuming, labor intensive, and difficult [18], and is often inappropriate for endangered species. However, several recent studies have used high-throughput sequencing to explore parasite diversity. For example, Tanaka et al. [19] successfully used high-throughput 18S rDNA sequencing to analyze worm diversity. Li et al. [20] also applied high-throughput sequencing to detect parasite diversity in yaks from the Gannan Tibetan Autonomous Prefecture. Wylezich et al. [21] used untargeted metagenomics to simultaneously identify protists and helminths in pre-diagnosed fecal and tissue samples. Porazinska et al. [22] successfully applied high-throughput sequencing to assess species-level diversity of nematodes in environmental samples through a set of control experiments. In addition, Porazinska et al. [23] analyzed the composition and structure of nematode communities using high-throughput sequencing reads generated from small subunit (SSU) diagnostic loci.

Given their vulnerability, any disease could increase the risk of extinction in crocodile lizards [11]. In this study, to investigate unexplained crocodile lizard deaths, we use high-throughput sequencing to detect intestinal parasites and explore potential differences in parasitic infections between captive and wild populations. We also compare parasitic infections in crocodile lizards from different regions to ascertain whether parasitic diseases exhibit regional differences and to implement different control measures depending on the infection status in different regions.

## 2. Materials and Methods

### 2.1. Ethics Statement

All experimental procedures were conducted following the guidelines approved by the Institutional Animal Care and Use Committee (IACUC) of Guangxi Normal University (Reference Number: 202209-003).

### 2.2. Sample Collection

In July 2021, fecal samples from 44 crocodile lizards were collected from Guangxi Daguishan Crocodile Lizard National Nature Reserve (24°08′ N, 111°41′ E), and fecal samples from 25 crocodile lizards were collected from Guangdong Luokeng *S. crocodilurus* National Nature Reserve (24°31′ N, 113°21′ E). The 69 crocodile lizards were separated into three groups, i.e., a wild group from Beilou Field Station in Daguishan National Nature Reserve (PBLW, *n* = 13), a captive group from Gandong Breeding Station in Daguishan National Nature Reserve (PGDB, *n* = 31), and a captive group from Luokeng Nature Reserve (PLK, *n* = 25). During the same period, we also collected four soil samples, four food samples (three earthworms and one cricket), and two livestock manure samples (fed to earthworms) from the Gandong Breeding Station to examine the sources of parasites.

All fecal samples were taken from the gut of the crocodile lizards using a Puritan Calgiswab Sterile Urogenital Calcium Alginate Sampler 25-801A50 (Puritan Medical Products Company LLC, Guilford, ME, USA) measuring 14 cm. After collection, the fecal samples were transported to the lab for DNA extraction within 24 h. The fecal samples were stored in a refrigerator at −80 °C before DNA extraction.

### 2.3. DNA Extraction

DNA extraction and sequencing were conducted by the Majorbio Corporation (Shanghai, China), according to established protocols. Total DNA was extracted from fecal samples using an EZNA^®^ Soil DNA Kit (Omega Bio-Tek, Norcross, GA, USA). We pulverized the fecal samples and added buffer, and then used magnetic beads to extract DNA. Genomic DNA was extracted from soil samples using the Omega^®^ Soil DNA Kit D5625 (Omega Bio-Tek, Norcross, GA, USA). The soil components were leached with sterile water to obtain a supernatant, which was then treated with a specially formulated buffer-containing detergent and proprietary cHTR Reagent. The binding conditions were then adjusted, and the sample was applied to an HiBind^®^ DNA Mini Column. Pure DNA was eluted in low ionic strength buffer. Genomic DNA was extracted from the animal tissue using an Omega^®^ Insect DNA Kit D0926 (Omega Bio-Tek, Norcross, GA, USA). Animal tissue was thoroughly ground, leached with sterile water, lysed in a high salt buffer containing CTAB (Hexadecyl trimethyl ammonium Bromide), and extracted with chloroform. Following rapid alcohol precipitation, the binding conditions were adjusted, and DNA was further purified using HiBind^®^ DNA Mini Columns. The purified DNA was used for polymerase chain reaction (PCR), restriction digestion, and next-generation sequencing. Extracted DNA integrity was tested using 1.0% agarose gel electrophoresis.

### 2.4. PCR Amplification and DNA Sequencing

To reduce the effect of primer bias, two primer sets were used; the first primer set was TAReuk454FWD1F (5′-CCAGCASCYGCGGTAATTCC-3′) and TAReukREV3R (5′-ACTTTCGTTCTTGATYRA-3′) [24], and the second primer set was Entam1F (5′-GTTGATCCTGCCAGTATTATATG-3′) and Entam3R (5′-GCTGCCTTCCTTAGAAGTGGT-3′) [25]. For sequencing, we built a 20 µL reaction system using TransGen AP221-02: TransStart FastPfu DNA polymerase (TransGen Biotech, Beijing, China), including 5×FastPfu Buffer (4 μL), 2.5 mM dNTPs (2 μL), forward primer (5 µM, 0.8 µL), reverse primer (5 µM, 0.8 µL), FastPfu polymerase (0.4 μL), bovine serum albumin (BSA, 0.2 µL), template DNA (10 ng), and distilled water (to volume). The PCR products were analyzed on a 2% agarose gel stained with ethidium bromide (EB) following electrophoresis. Purification of PCR products was performed using an AxyPrep DNA Gel Extraction Kit (Axygen, Tewksbury, MA, USA). The sequencing library was prepared using a NextFlex^®^ Rapid DNA-Seq Kit (Bioo Scientific, Austin, TX, USA). High-throughput sequencing was performed using the commercial Illumina MiSeq platform (150-bp paired-end reads) (Majorbio, Shanghai, China).

### 2.5. Data Analysis

After sequencing, operational taxonomic units (OTUs) were constructed, and raw tags were filtered using the QIIME2 package (v2020.8) to remove low-quality and chimeric sequences. Sequences with ≥97% similarity were assigned to the same OTU using UPARSE (v7.1) [26,27]. Representative sequences for each OTU were annotated using the RDP classifier [28] and BLAST by searching the SILVA database (https://www.arb-silva.de/) (accessed on 12 October 2021) (threshold = 0.7) and the NT database (ftp://ftp.ncbi.nih.gov/blast/db/) (accessed on 12 October 2021) (threshold = 0.7). The community species composition of each sample was calculated at each taxonomic level (domain to species). An OTU abundance table was used for visualization and analysis. All raw sequences obtained from high-throughput sequencing were deposited in the NCBI Sequence Read Archive (SRA) under accession number PRJNA853948.

Rarefaction curves were produced by the analytical expression of Mao et al. [29] and Colwell et al. [30] using EstimateS v8.2 [31]. Sample-based rarefaction is a powerful tool for assessing species richness based on equal-size sampling approaches [32]. Rarefaction curves can be used to evaluate species abundance in samples with varying sequencing data, and to determine whether sequencing data are adequate [33]. A flat rarefaction curve indicates that sequencing data are reasonable, with additional data only yielding a low number of new OTUs; conversely, a non-flat curve indicates that continued sequencing may generate new OTUs.

Parasite-infected samples were filtered from the species classification annotation table using GraphPad Prism v8.0.1, and then the species and cases of parasites infected in each sample were counted. Samples with a single parasite infection were categorized as single infections, whereas those with two or more parasites infections were categorized as mixed infections. The parasitic infection rate of crocodile lizards was analyzed using the chi-square test and Fisher’s exact test in GraphPad Prism v8.0.1 and R v3.6.3 (stats package), and differences were considered significant at *p* < 0.05.

## 3. Results

### 3.1. Observed Rarefaction Curves

**Figure 1:**
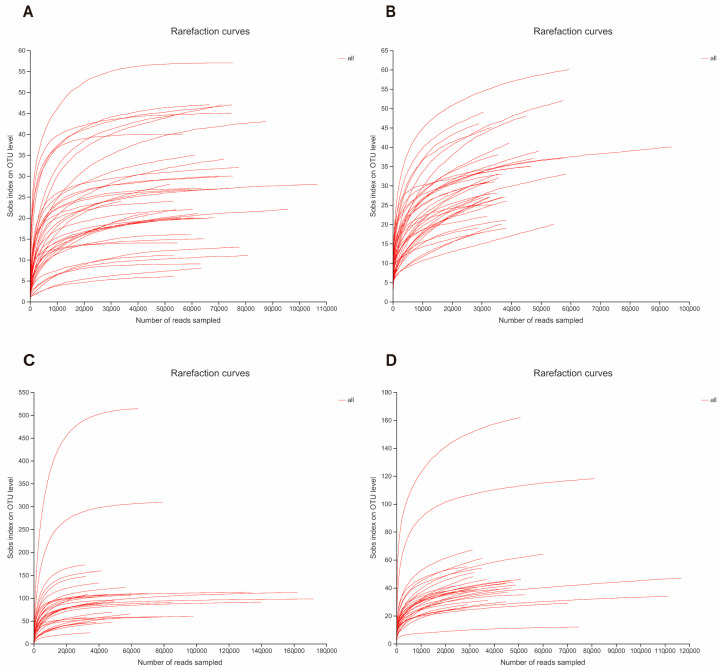
Rarefaction curve of each sample. (**A**,**B**) represent the rarefaction curves of 38 samples from Luokeng and Beilou. (**C**,**D**) represent the rarefaction curves of 31 samples from Gandong.

Sample-based rarefaction curves for all samples are shown in Figure 1, where A and B represent the rarefaction curves of 38 samples from Luokeng and Beilou; and C and D represent the rarefaction curves of 31 samples from Gandong. The results indicate that species richness (i.e., OTU number) in the samples increased with the increase in sequencing depth (Figure 1). However, as sequencing depth (total number of sequences) continued to increase, the rarefaction curves flattened and remained constant (Figure 1). Therefore, the smooth rarefaction curves indicated that our sample size was sufficient and parasite sequencing was reliable in terms of depth and accuracy.

### 3.2. Analysis of Parasite Community Composition

**Figure 2:**
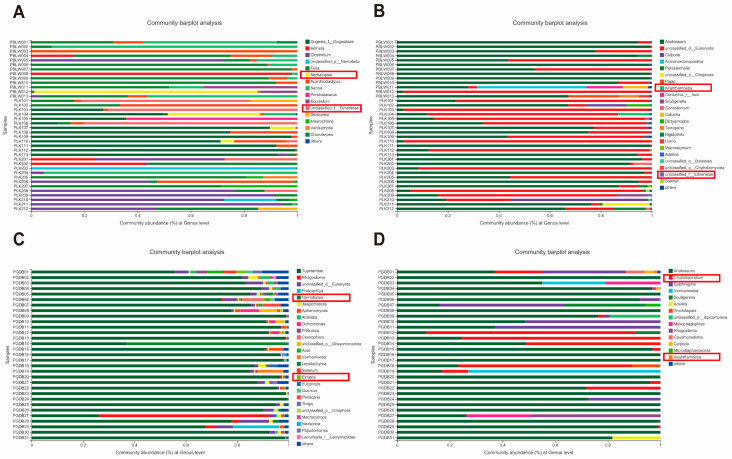
Composition of parasites in each sample at genus level. Different colors indicate different groups, with details shown on right sides of each figure, respectively. (**A**,**B**) show composition of parasites in Luokeng and Beilou. (**C**,**D**) show composition of parasites in Gandong. Parasites discovered in the current study are contained within rectangle.

At the phylum level, Apicomplexa was detected in all three crocodile lizard groups, while Nematoda was only found in the PLK captive crocodile lizards (Figure S1). At the class level, Colpodea, Conoidasida, and Spirotrichea were found in all three crocodile lizard groups, while Litostomatea was only found in the PGDB captive crocodile lizards (Figure S2). At the order level, Eucoccidiorida was detected in the PGDB and PLK captive crocodile lizards; and Trombidiformes and Longamoebia were only found in the PLK captive crocodile lizards (Figure S3). At the family level, Eimeriidae and Acanthamoebidae were observed in both PLK and PGDB captive crocodile lizard groups; Cryptosporidiidae was only found in the PGDB captive crocodile lizards (Figure S4). At the genus level, *Nematopsis* was found in all three crocodile lizard groups (Figure 2A,C); *Eimeria* and *Acanthamoeba* were observed in the PLK and PGDB captive crocodile lizards (Figure 2), and *Cryptosporidium* was only observed in the PGDB captive crocodile lizards (Figure 2D).

### 3.3. Analysis of Parasitic Infection

Parasitic infection in the 69 crocodile lizard samples was analyzed (Table 1), resulting in the detection of 23 parasite-positive fecal samples. The total parasitic infection rate was 33.33% (23/69). Four parasites were found in the samples, including *Cryptosporidium* (17.39%, 12/69), *Eimeria* (15.94%, 11/69), *Nematopsis* (7.25%, 5/69), and *Acanthamoeba* (4.35%, 3/69). The different parasite species showed significant differences in infection rates (χ^2^ = 8.54, *p* < 0.05, chi-squared test). The parasitic infection rates of crocodile lizards in Gandong, Luokeng, and Beilou were 45.16% (14/31), 32.00% (8/25), and 7.69% (1/13), respectively, which were not significantly different (χ^2^ = 5.82, *p* > 0.05, chi-squared test). Multiple parasites were detected in seven fecal samples (six samples were infected with two parasites, and one sample was infected with all three), with a mixed infection rate of 30.43% (7/23), while the single infection rate was 69.57% (16/23). *Cryptosporidium* and *Eimeria* were most common in mixed infections, while in single infections, *Cryptosporidium* was the main infection type.

The total parasitic infection rates in the captive breeding population (39.29%, 22/56) and wild population (7.69%, 1/13) were significantly different (*p* < 0.05, Fisher’s exact test, Table 2). All four parasitic infection rates were higher in the captive population than in the wild population (Table 2), although the differences were not significant (*p* > 0.05, Fisher’s exact test).

The parasitic infection rates of crocodile lizards in the Daguishan and Luokeng nature reserves were 34.09% (15/44) and 32.00% (8/25), respectively (Table 3), which were not significantly different (*p* > 0.05, Fisher’s exact test). *Eimeria*, *Nematopsis,* and *Acanthamoeba* were detected in both regions, although *Nematopsis* and *Eimeria* infection was higher in Luokeng and *Acanthamoeba* infection was higher in Daguishan (Table 3). *Cryptosporidium* was only detected in Daguishan (infection rates of 27.27%) (Table 3). Statistical analysis using the fisher.test function in R package (v3.6.3) showed that the species found in the two reserves differed significantly (*p* < 0.01, Fisher’s exact test). Only *Cryptosporidium* infection showed a significant difference between the two regions (*p* < 0.01, Fisher’s exact test). Thus, *Cryptosporidium* infection varied by geographical region, with crocodile lizards from Daguishan showing higher susceptibility to *Cryptosporidium* infestation.

### 3.4. Source of Crocodile Lizard Parasites

**Figure 3:**
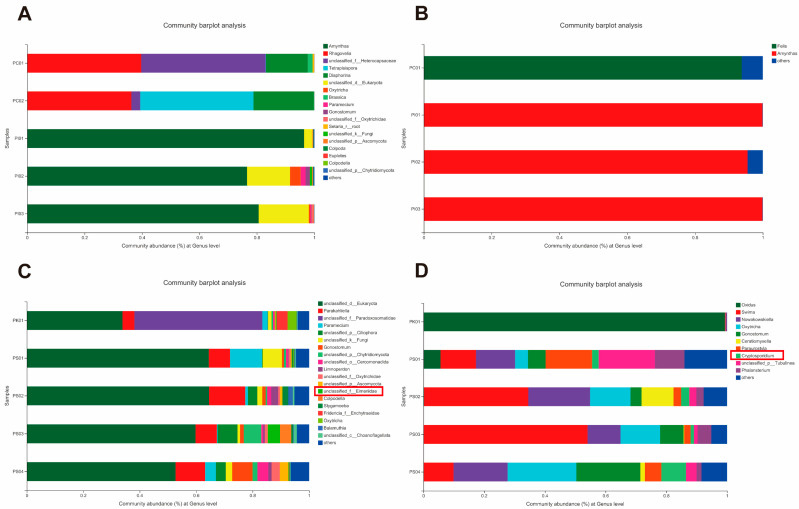
Composition of parasites in environmental samples at genus level. Different colors indicate different groups, with details shown on right side of each figure, respectively. PC01 and PC02 represent livestock manure samples (fed to earthworms); PI01, PI02, and PI03 represent earthworm food samples; PK01 represents cricket food sample; PS01, PS02, PS03, and PS04 represent soil samples. (**A**,**B**) show composition of parasites in livestock manure and earthworm food samples. (**C**,**D**) show composition of parasites in cricket food and soil samples. Parasites discovered in the current study are contained within rectangle.

To trace the source of parasites, four soil samples, four food samples (three earthworms and one cricket), and two livestock manure samples (fed to earthworms) from the crocodile lizard living environment were sequenced (Figure 3). The results show that *Cryptosporidium* was found at low relative abundance in all soil samples, and Eimeriidae was found at very low relative abundance in all soil samples and the cricket sample. However, no other parasites were detected in the above samples.

## 4. Discussion

In the current study, we investigated parasitic infections in crocodile lizards to elucidate the potential cause of unexplained deaths. The results indicate that parasitic infection was significantly higher in the captive-bred crocodile lizards than in the wild population. Additionally, the species found in the two reserves differed, and only *Cryptosporidium* infection showed a significant difference between Daguishan and Luokeng. Based on analysis of parasite sources, several parasites were detected at very low levels in the soil and food samples.

Captive crocodile lizard infection and disease have become more common in recent years, placing considerable strain on conservation efforts. To clarify the cause of unexpected deaths, Jiang et al. [11] and Xiong et al. [6] successively investigated two distinct skin diseases in crocodile lizards, revealing serious bacterial infections and probable co-infection. Increasing evidence suggests that co-infection of parasites with viruses, bacteria, or other parasites can impact host immunoreactivity and illness outcomes [34,35,36]. Several reports suggest that co-infection with bacteria and malaria parasites worsens clinical outcomes in patients, including respiratory distress, anemia, and mortality [37,38]. Parasitic infections can suppress inflammatory responses, which can, in turn, affect illness outcomes caused by other viral, bacterial, or parasitic infections [36]. Based on our results, the incidence of parasitic infection was significantly higher in the captive crocodile lizards than in the wild population, which warrants further attention. This finding suggests that captive crocodile lizard populations may be more susceptible to parasitic diseases. According to previous research, captive animals tend to show lower disease resistance and greater disease sensitivity [39,40], consistent with our results. Although breeding enclosures are designed to provide proper physical conditions, captive animals inevitably experience chronic stress, which can lead to immunosuppression and disease [9]. Parasites can rapidly spread from one individual to multiple individuals under high-density captive conditions [41,42]. Additionally, compared to the captive crocodile lizards, the sample size was smaller in the wild crocodile lizards, and thus may be more likely to contain non-infected or slightly infected individuals, while missing the rarer heavily infected individuals [43]. Furthermore, the Beilou area is a relatively enclosed environment [44], with wild crocodile lizards minimally exposed to the outside world and thus less likely to be infected with parasites. These reasons may explain why the wild population in Beilou had a lower parasitic infection rate than the captive population. Previous research on skin nodule infection in crocodile lizards found that samples infected with *Austwickia chelonae* were co-infected with *Salmonella* sp., *Acinetobacter* sp., *Pseudomonas* sp., and *Halomonas* sp. [11]. Additionally, the co-infection of *Pseudomonas aeruginosa* and *Saprolegnia ferax* has also been observed in several severe skin ulcer cases in crocodile lizards [6]. In contrast to skin samples [6,11], parasite composition in fecal samples was primarily based on single infection. However, as skin infections are common in crocodile lizards, with parasites infecting lizards through skin wounds, then migrating through host tissues or feeding on intestinal epithelial cells [45], we speculate that crocodile lizard mortality may be due to bacterial and parasitic co-infection. When reptiles are under conditions of stress, such as parasitism, trauma, or other infectious disease processes, *Salmonella* spp. can penetrate the intestinal mucosal barrier and initiate systemic illness [9]. Furthermore, *Salmonella* can spread from the skin to internal organs [11], potentially leading to the death of crocodile lizards. Co-infection of *Clostridium perfringens* with *Eimeria maxima*, a protozoan parasite that lives in the gut and causes coccidiosis in poultry, is linked to necrotic enteritis [46]. In our earlier research, intestinal inflammation was identified as a common factor in some crocodile lizard deaths [6], likely due to *Salmonella* and *Eimeria* co-infection. Xiao et al. [47] also reported that *Cryptosporidium saurophilum*, an intestinal parasite found in lizards, does not appear to affect adult lizards, but has been shown to cause weight loss, abdominal swelling, and mortality in some colonies of juvenile geckos (*Eublepharis macularius*). Due to the strong host specificity of *Cryptosporidium* [48], *Cryptosporidium saurophilum* may also cause intestinal abnormalities in juvenile crocodile lizards, and co-infection with bacteria may lead to death. However, this needs to be further clarified through species characterization, which is the next stage of our research.

Our findings reveal that parasite species and number varied in the two nature reserves, with higher species and number in Daguishan than in Luokeng. Given the similar natural environments of the two regions [49,50], it is possible that other factors contributed to the differences between the two regions. For example, intrinsic host factors (e.g., immune system, age, and sex) may influence the probability of a parasite infecting a suitable host [51]. Some filaria and nematodes are transmitted by arthropod vectors [52]. Crocodile lizards are sit-and-wait predators, and their food composition is largely dependent on the species and number of invertebrates in the surroundings [49]. In the wild, earthworms are a primary food resource for crocodile lizards, but they also consume other insects, including locusts and crickets, and spiders, whereas captive crocodile lizards are raised on a mixed diet of earthworms, crickets, and mealworms [49,53]. Consequently, crocodile lizards may become infected with parasites due to the consumption of certain arthropods (e.g., crickets, spiders, and mealworms). Additionally, different sanitation and rearing conditions, as well as the health and immune status of animals, can affect the presence and infectivity of parasites [54], potentially contributing to crocodile lizard infection. At the same time, difference in sample size between the two regions may affect the sensitivity and accuracy of the results [54,55]. In addition, captive crocodile lizards are usually raised in relatively small (2 × 3 m) enclosures, with the main water supply coming from nearby streams (e.g., Hejiang River in Daguishan and Beijiang River in Luokeng) [6,50,56]. In lotic-dominated freshwater ecosystems, the combined effects of unidirectional water flow and mobility of the most mobile host are primary drivers of parasite distribution, especially those with complex life cycles (e.g., *Cryptosporidium*) [57,58]. *Acanthamoeba* exposure occurs frequently through water contact [59] and understanding the organisms present in the water supply is important for preventing crocodile lizard diseases. Furthermore, climate change may cause geographical and phenological shifts, as well as alteration in the dynamics of parasite transmission, increasing the potential for host switching [60]. At the same time, the risk of vector-transmitted diseases may increase in regions where environmental conditions are altered by climate change [61,62].

*Cryptosporidium* is a zoonotic gastrointestinal parasite, which can infect a broad range of hosts [63,64]. *Cryptosporidium* infection among reptiles mainly presents as a gastroenteritis-like syndrome [65,66,67] and disease severity ranges from mild to severe, with symptoms varying based on the site of infection and the host’s dietary and immunological state [65]. *Cryptosporidium* infection is primarily spread by direct contact with human or animal excrement, or indirectly through a transmission medium such as contaminated water, food, or pollutants [68]. According to our results, only captive crocodile lizards in Guangxi Daguishan Nature Reserve carried *Cryptosporidium*. This may be linked to the inadequate cleaning and disinfection of the rearing enclosures or to close contact with keepers. The life cycle of *Cryptosporidium* is completed within a single host, followed by the excretion of many infectious oocysts in fecal matter [69]. Consequently, crocodile lizard feces should be removed, and enclosures disinfected as quickly as possible. In addition, clean water is critical for crocodile lizard survival. Infective *Cryptosporidium* oocysts are environmentally robust, not only small enough to pass through physical barriers in water treatment but also resistant to many of the disinfectants used [69]. Thus, for captive crocodile lizards, water needs to be changed regularly, water treatment needs to be enhanced, and water health needs to be monitored. Additionally, to reduce spillover risk, breeders should sanitize all areas before handling lizards and take protective measures (e.g., gloves, masks, and protective clothing) during lizard operations. Previous studies have shown that carnivorous snakes and lizards infected with *Cryptosporidium* oocysts, possibly from the ingestion of infected rodents, may be asymptomatic [70]. Furthermore, predator–prey transmission of *Cryptosporidium* may occur, with *Cryptosporidium*-infected food maintaining a source of oocysts that are passively transferred through snakes [71]. As such, *Cryptosporidium* oocysts can be detected in lizards and snakes even when they are not infected. Therefore, the *Cryptosporidium* species found in crocodile lizards may have originated from prey, and the *Cryptosporidium* oocysts may have been passively transferred through the crocodile lizard without actual infection.

*Cryptosporidium* and Eimeriidae were detected in all soil samples collected onsite, as well as in fecal samples collected from the captive crocodile lizards, indicating that *Cryptosporidium* and *Eimeria* may come from the soil in the living environment. At the same time, Eimeriidae was also detected in the cricket sample collected onsite; however, soil samples had a higher relative abundance of Eimeriidae compared to the cricket sample. Thus, for captive crocodile lizards, environmental disinfection may be a viable disease prevention strategy. In addition, no parasites were detected in the earthworm samples, suggesting that earthworms may be a superior food choice for crocodile lizards, as reported in previous research [7]. *Nematopsis* and *Acanthamoeba* were not found in any of the food, soil, or livestock manure samples (fed to earthworms). Jiang et al. [7] reported that high concentrations of potentially pathogenic bacteria in loach diets may increase the chance of crocodile lizard infection. Additionally, crocodile lizards often compete for territory, mates, and food, which can cause trauma and provide an opportunity for parasitic infection [11]. Regrettably, we did not collect water from the sampling sites or analyze other factors. Consequently, parasites may potentially infect crocodile lizards via water, other food (loaches), or other means (skin wounds). Further research is needed to determine the actual source of parasites. For parasitic control, first and foremost, comprehensive cleaning and disinfection programs are required in captivity [9]. Secondly, reducing captive density should help to decrease disease spread [6]. Previous studies have also shown that dietary supplementation may lessen parasitism, e.g., urea and fishmeal reduced parasitism levels in *Toxoplasma*-infected sheep by 30% and 44%–99%, respectively, while protein supplementation reduced nematode levels in infected calves by 40% [72]. Similarly, improving crocodile lizard nutrition may enhance immunity (resistance) and increase resilience.

## 5. Conclusions

This study provided an overview of parasitic infections in captive and wild crocodile lizards. From a disease prevention perspective, these findings have great significance for crocodile lizard conservation. However, due to the limitations of clinical samples and experimental conditions, the parasites were not characterized at the species level, and the pathogenic mechanism of intestinal parasites in crocodile lizards still requires further exploration.

## Figures and Tables

**Table 1:** Parasitic infection of crocodile lizards at different sampling sites.

				Infection Rate of Different Parasites (%)			
Sample Site	Sample Number	Positive Sample Number	Infection Rate (%)	*Eimeria*	*Cryptosporidium*	*Nematopsis*	*Acanthamoeba*
Gandong	31	14	45.16	16.13 (5/31)	38.71 (12/31)	3.23 (1/31)	6.45 (2/31)
Luokeng	25	8	32.00	24.00 (6/25)	-	12.00 (3/25)	4.00 (1/25)
Beilou	13	1	7.69	-	-	7.69 (1/13)	-
Total	69	23	33.33	15.94 ^a^ (11/69)	17.39 ^a^ (12/69)	7.25 ^a^ (5/69)	4.35 ^a^ (3/69)

Note: (1) -: No positive samples detected. (2) ^a^ Comparison between four parasite species, *p* < 0.05, confidence interval is 95%.

**Table 2:** Parasitic infection of crocodile lizards in different populations.

				Infection Rate of Different Parasites (%)			
Population	Sample Number	Positive Sample Number	Infection Rate (%)	*Eimeria*	*Cryptosporidium*	*Nematopsis*	*Acanthamoeba*
Captive	56	22	39.29 ^a^	19.64 (11/56)	21.43 (12/56)	7.14 (4/56)	5.36 (3/56)
Wild	13	1	7.69 ^a^	-	-	7.69 (1/13)	-

Note: (1) -: No positive samples detected. (2) ^a^ comparison between captive and wild populations, *p* < 0.05, confidence interval is 95%.

**Table 3:** Parasitic infection of crocodile lizards at different localities.

				Infection Rate of Different Parasites (%)			
Locality	Sample Number	Positive Sample Number	Infection Rate (%)	*Eimeria*	*Cryptosporidium*	*Nematopsis*	*Acanthamoeba*
Daguishan	44	15	34.09	11.36 (5/44)	27.27 ^a^ (12/44)	4.55 (2/44)	4.55 (2/44)
Luokeng	25	8	32.00	24.00 (6/25)	-	12.00 (3/25)	4.00 (1/25)

Note: (1) -: No positive samples detected. (2) ^a^ Comparison between two localities, *p* < 0.01, confidence interval is 95%.

## Data Availability

Raw sequence data were submitted to the NCBI Sequence Read Archive (SRA) under accession number PRJNA853948.

## Supplementary Materials

The following supporting information can be downloaded at: https://www.mdpi.com/article/10.3390/ani12202726/s1. Figure S1: Composition of parasites in each sample at the phylum level; Figure S2: Composition of parasites in each sample at the class level; Figure S3: Composition of parasites in each sample at the order level; Figure S4: Composition of parasites in each sample at the family level.
