# Supplementary material for: Investigation of Parasitic Infection in Crocodile Lizards (Shinisaurus crocodilurus) Using High-Throughput Sequencing
Source: Animals (Basel). 2022 Oct 11;12(20):2726. doi: 10.3390/ani12202726 (PMC9597849; doi:10.3390/ani12202726)
Supplement: Supplementary file 1 [file animals-12-02726-s001.zip › animals-1926438-supplementary.pdf]

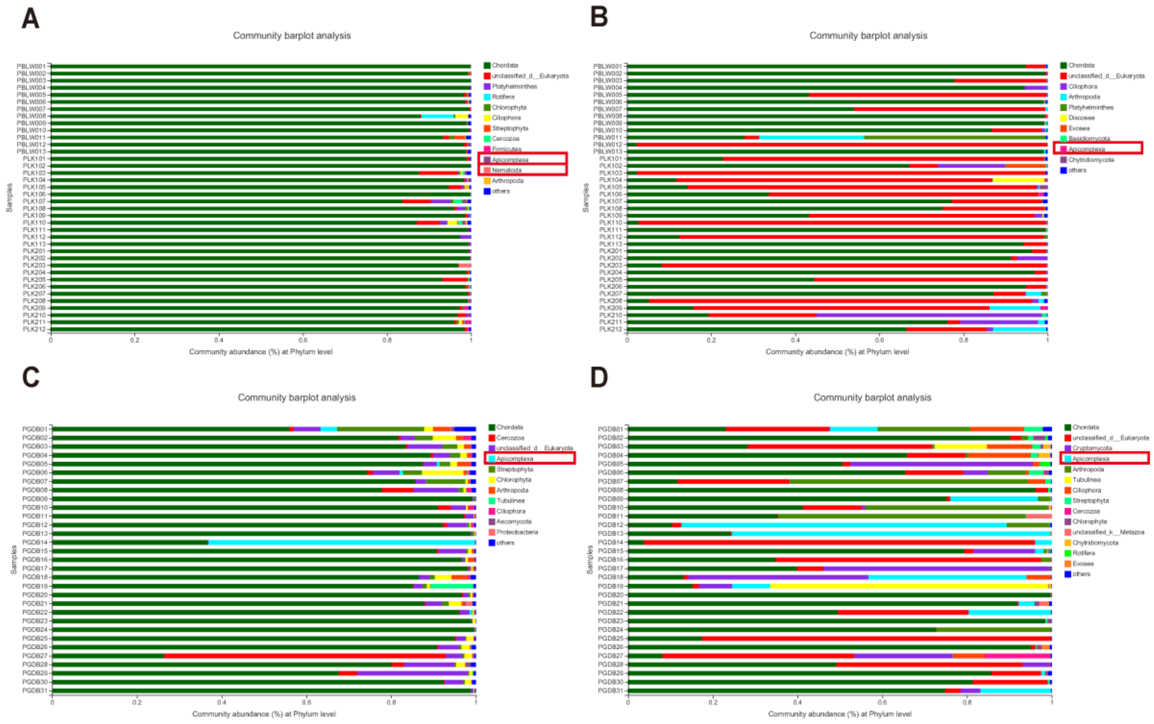

Figure S1. Composition of parasites in each sample at phylum level.

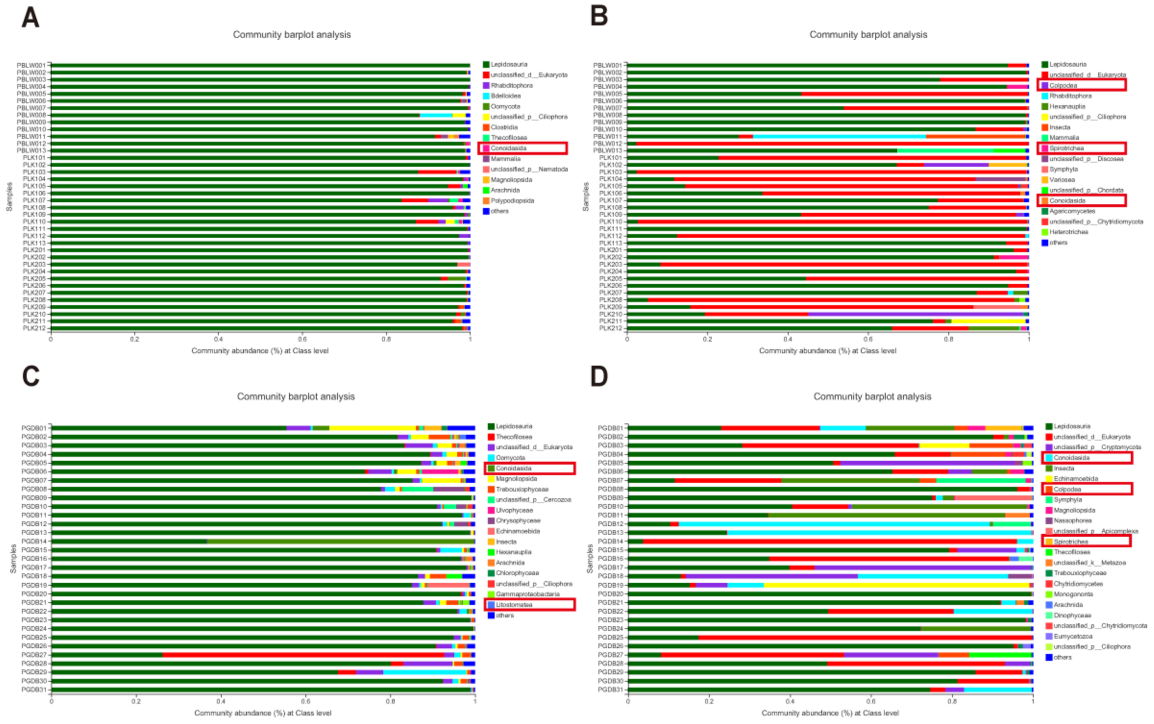

Figure S2. Composition of parasites in each sample at class level.

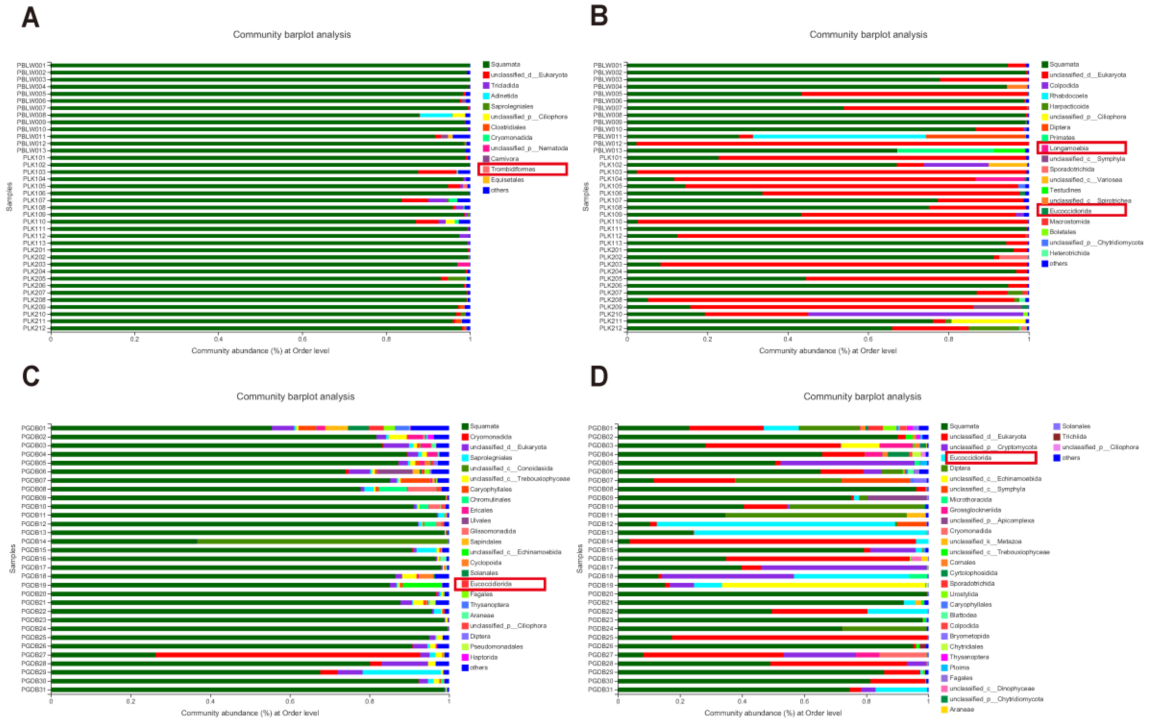

Figure S3. Composition of parasites in each sample at order level.

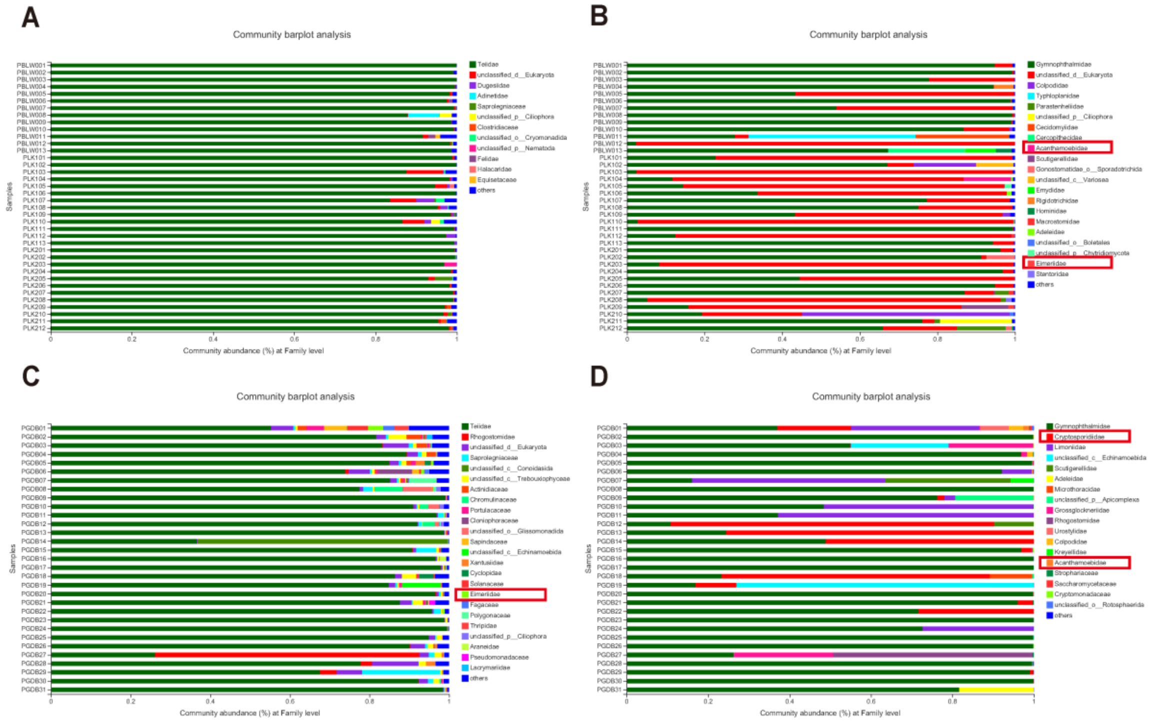

Figure S4. Composition of parasites in each sample at family level.
